# Metabolic syndrome related health inequalities in Korean elderly: Korean National Health and Nutrition Examination Survey (KNHAES)

**DOI:** 10.1186/s12939-014-0097-z

**Published:** 2014-11-19

**Authors:** Hak-Seon Kim

**Affiliations:** Department of Foodservice Management, Kyungsung University, 309 Suyeong-ro Nam-gu, Busan, 608-736 South Korea

**Keywords:** Metabolic syndrome, Dietary patterns, Body image, Muscle and fat mass

## Abstract

While the prevalence of metabolic syndrome is increasing, little is presently known about this syndrome in Korean elderly. This study aimed to group metabolic risk factors and to examine the associations between groups of health living conditions and metabolic syndrome using data from the Korean National Health Examination and Nutritional Assessment (KNHANES). A total of 1,435 subjects aged over 65 years old with both biochemical and dietary data information were obtained from the 4th and 5th KNHANES (2007–2012). Using stratified and multistage probability sample data, five components of metabolic syndrome were adopted to identify health inequalities. Our findings show that groups of health living conditions such as dietary pattern, body image, muscle mass, and fat mass were differentially associated with metabolic syndrome risk factors. Future studies are necessary to examine the underlying mechanisms of individual health living conditions to better understand the role of metabolic risk factors in metabolic syndrome in elderly.

## Introduction

Rapid economic growth and adoption of a Western lifestyle over the last decade in Korea have led to changes in dietary habits, such as frequent dining out and consumption of fast food, especially among Korean elderly. According to the Korean National Health Examination and Nutrition Survey (KNHANES) in 2012, the percentage of who dined out at least once a day was 36.7% and who skipped breakfast was 27.6% [1]. The percentages of those consuming carbonated drinks and fast food more than three times a week were as high as 26.3% and 12.3%, respectively. Adolescents in the Minnesota study reported similar trends, in that consumption of soft drinks and fast food increased, but that of fruits, vegetables, or milk decreased, and their preference for fast food or soft drinks may lead to nutritional imbalances. These undesirable dietary habits affect the health status [2].

In addition, there is growing awareness about the association of dietary habit and health risk factors. A higher risk of having metabolic syndrome (MS) is associated with higher body mass index (BMI), older age, and lifestyle factors including low physical activity and smoking [3]. Although there is currently no universally accepted definition for MS, its prevalence is increasing. Using data from KNHANES, the prevalence of metabolic syndrome increased from 4.0% in 1998 to 5.9% in 2001, 6.6% in 2005, and 7.8% in 2007 [4]. Recently, due to the arbitrary definitions of metabolic syndrome, several studies were conducted to structuralize the clustering phenomenon of the individual components of complex metabolic syndrome [5-8]. In these studies, three or four patterns of metabolic syndrome, including insulin resistance, obesity, dyslipidemia and hypertension, were suggested. To understand more fully the etiology of metabolic syndrome, determining these individual patterns is necessary to examine their relationships with other factors.

A few studies have examined the association of dietary factors with metabolic syndrome or its risk factors in Korean elderly. Therefore, assessment of the health related factors on metabolic abnormalities may need simultaneous consideration of these covariates. The objective of this study was to examine the impact of health related factors such as dietary patterns, body image, appendicular skeletal muscle mass, and fat mass on the risk of having the MS and IR (Insulin Resistance) after controlled for other covariates including lifestyle factors in normal-weight and obese men and women using a nationally representative sample of Korean elderly using data from the Korean National Health and Nutrition Examination Survey.

## Methods

### Data collection and study population

The data in this study were obtained from the fourth and fifth Korean National Health and Nutrition Examination Surveys (KNHANES 2007–2012). Details of the KNHANES performed in 2007–2012 have been previously described [4]. In brief, health examination, dietary measurement, height, and weight were obtained using standardized techniques and calibrated equipment. Body mass index (BMI) was calculated by dividing weight (kg) by height (m^2^). Blood pressure was measured using a sphygmomanometer with the subject in a sitting position. Three measurements were taken at 5-min intervals in the morning after having fasted for at least 8 hour, and the average of the second and third measurements was used. Fasting glucose, fasting insulin, total cholesterol, triglycerides (TG), and high-density lipoprotein (HDL) were analyzed in a central, certified laboratory. A dual-energy X-ray absorptiometry (DXA) scan was performed to measure total body fat mass, total body fat percentage, and lean mass using fan-beam technology (Lunar Corp., Madison, WI). The body fat percentage of a human being is the total mass of fat divided by total body mass. A general questionnaire was administered to assess basic demographic and health-related information. Dietary intake was measured by the single 24-hour dietary recall method and compared with the Recommended Daily Allowance (RDA). Trained staff instructed the respondents to recall and describe all the foods and beverages they had consumed in the previous day. Food models and measuring bowls, cups, and spoons were used to assist in estimating portion sizes. Among 6,453 eligible elderly aged over 65 years, those who had participated in both the health examination and dietary survey were selected (n = 1,598). We excluded those subjects who reported implausible energy intakes (<500 or ≥5,000 kcal per day) (n = 85), treatment of diabetes (n = 13), fasting for less than 8 hours (n = 183), or missing values for blood or target variables (n = 1,317). Finally, a total of 1,435 subjects were analyzed in this study.

### Definition of main variables

In this study, we applied group five components of metabolic syndrome, including waist circumference, and fasting blood glucose levels, HDL-cholesterol, triglycerides, and systolic and diastolic blood pressure. Three identified dietary patterns and three obesity groups were adopted according to their intake nutrients and their body image. In addition, subjects were categorized into quartiles by pattern score of ASM and fat mass. Obesity classification was determined according to the BMI criteria established by the Obesity Task Force (IOTF), World Health Organization (WHO) and the Korean Society for the Study of Obesity (KSSO) [9]. Appendicular skeletal muscle mass (ASM, kg) was defined as the sum of lean soft tissue mass in the arms and legs following the method of Oh et al. [3]. We calculated ASM as a percentage of body weight (Wt), modifying methods published Lim et al. [10].

The definition of metabolic syndrome in elderly used in this study was adopted from the modified version of the National Cholesterol Education Program Adult Treatment Panel III (NCEP-ATPIII) [11]. Metabolic syndrome was diagnosed if more than three of the following five categories were satisfied: 1) waist circumference ≥90th percentile (based on gender-specific percentiles by age according to the Korean growth chart, 2007 [12]), 2) fasting blood glucose level ≥100 mg/dL 3) serum HDL-cholesterol level ≤40 mg/dL, 4) serum triglyceride level ≥110 mg/dL, and 5) systolic or diastolic blood pressure ≥90th percentile [13].

### Statistical analysis

All statistical analyses were conducted using SPSS version 20.0 (SPSS, IBM, NY, USA). The level of significance was set at p <0.05. For pattern analysis, results of previous study were adopted. Since the pattern analysis can be affected by differences in scales among indicators, the five risk factors of waist circumference, triglycerides, systolic and diastolic blood pressure, HDL-cholesterol and fasting blood glucose were standardized. The number of factors was determined based on the eigenvalue, scree test, and the interpretability of the derived factors [14]. Basic characteristics were tested using chi-square tests according to the dietary pattern groups. Logistic regression was used to calculate odds ratios (ORs) with 95% confidence intervals (CIs) for the body composition traits. All models were adjusted for age, gender, education, smoking, and physical activity in all logistic regression models [15].

## Results

Gender, subjective body image, obesity, whole body fat mass and waist circumference according to the dietary pattern groups are presented in Table 1. There are significant differences in gender, subjective body image, and whole body fat mass among 3 groups. Meat and alcohol dietary group showed higher level of subjective body image as overweight than other two groups. In accordance with previous study, there are significant associations between dietary patterns and gender and Westernized Korean dietary group showed higher level of whole body fat mass. Previous study revealed that gender-related differences in relation to alcohol consumption between males and females were detected [16]. Odds ratios for Metabolic syndrome factors by dietary patterns were presented in Table 2. In metabolic syndrome factors, the ‘Meat & Alcohol’ pattern showed a 93% increased blood pressure and ‘Westernized Korean’ pattern showed a 32% increased blood pressure, compared with the ‘Traditional Korean’ pattern. Other factors were not associated with the dietary patterns.

**Table 1:** **Demographic characteristics by dietary pattern group**

	**Traditional Korean**	**Meat & alcohol**	**Westernized Korean**	***p*** ^**a**^
Subjective body image (%)				0.003
as overweight	27.15	33.33	27.15	
Gender (%)				0.001
Male	35.50	47.52	45.85	
Female	64.50	52.48	60.17	
Obesity (%)	28.81	39.01	33.01	0.075
Whole body fat mass (%)	28.61 ± 0.70	28.06 ± 1.07	29.77 ± 0.56	0.005
Waist circumference (cm)	82.67 ± 0.51	84.16 ± 0.67	83.61 ± 0.67	0.744

Note. ^a^p from chi-square test.

**Table 2:** **Odds Ratios (OR) for metabolic syndrome factors by dietary patterns**

	**Traditional Korean (n = 538)**	**Meat & Alcohol (n = 282)**		**Westernized Korean (n = 615)**		***p*** ^**a**^
	**OR**	**OR**	**95% CI**	**OR**	**95% CI**	
Waist circumference (cm)^a^	1.00	1.003	0.706-1.426	1.002	0.724-1.387	1.000
Fasting Glucose (mg/dl)	1.00	1.263	0.895-1.783	1.244	0.882-1.755	0.260
HDL-cholesterol (mg/dl)	1.00	0.867	0.438-1.717	1.364	0.781-2.382	0.326
TG (triglyceride) (mg/dl)	1.00	0.811	0.556-1.182	0.788	0.587-1.057	0.246
Blood Pressure (mm Hg)	1.00	1.927	1.233-3.014	1.323	0.882-1.755	0.017

Note. ^a^Based on multiple logistic regression analysis, with 3 categories of each dietary patterns.

The multivariate-adjusted ORs (95% CI) for metabolic syndrome are presented in Table 3. Members of the ‘overweight’ group showed a 790.8% higher likelihood of having waist circumference, a 1,886.1% higher likelihood of having higher HDL-cholesterol, and a 99.5% higher likelihood of having hypertriglyceridemia compared with the ‘underweight’ group. Members of the ‘normal’ group showed a 207.2% higher likelihood of having waist circumference, a 1,874.4% higher likelihood of having higher HDL-cholesterol, and a 190.5% higher likelihood of having hypertriglyceridemia compared with the ‘underweight’ group.

**Table 3:** **Odds Ratios (OR) for metabolic syndrome factors by body image (BMI)**

	**Underweight (n = 79)**	**Normal (n = 955)**		**Overweight (n = 502)**		***p*** ^**a**^
	**OR**	**OR**	**95% CI**	**OR**	**95% CI**	
Waist circumference (cm)^a^	1.00	3.072	2.004-4.711	8.908	0.908-8.900	<0.001
Fasting Glucose (mg/dl)	1.00	1.211	0.541-2.712	1.654	0.697-3.926	0.171
HDL-cholesterol (mg/dl)	1.00	19.744	2.602-147.828	19.861	2.300-171.475	0.017
TG (triglyceride) (mg/dl)	1.00	2.905	1.182-7.140	1.995	0.724-5.499	0.014
Blood Pressure (mm Hg)	1.00	1.454	0.512-4.132	1.375	0.467-4.046	0.779

Note.^a^Based on multiple logistic regression analysis, with 3 categories of each body images.

Tables 4 and 5 present metabolic syndrome components according to quartiles of Appendicular Skeletal Muscles and Fat Mass. Waist circumference was significantly different by the quartiles of Appendicular Skeletal Muscles after adjusting for age and gender. Waist circumference and fasting glucose were associated with quartiles of fat mass. Other abnormalities were not associated with those two body composition factors. Especially, members of the second quartile group in fat mass showed 524.3%, third quartile group showed a 652.9%, fourth group members showed 1,358.9% higher likelihood of waist circumference compared with members of the first quartile group in fat mass. Waist circumference increased markedly, while the fasting glucose level decreased among the quartile groups in fat mass percentage. Other risk factors were not significantly different across quartile groups, or no consistent trend was seen.

**Table 4:** **Odds Ratios (OR) for metabolic syndrome factors by quartiles of Appendicular Skeletal Muscles (ASM) mass as percentage of body**

	**Q1 (n = 148)**	**Q2 (n = 148)**		**Q3 (n = 147)**		**Q4 (n = 147)**		***p*** ^**a**^
	**OR**	**OR**	**95% CI**	**OR**	**95% CI**	**OR**	**95% CI**	
Waist circumference (cm)^a^	1.00	0.270	0.131-0.556	1.817	1.160-2.846	0.882	0.559-1.390	<0.001
Fasting Glucose (mg/dl)	1.00	0.768	0.408-1.444	0.990	0.588-1.664	0.791	0.477-1.312	0.543
HDL-cholesterol (mg/dl)	1.00	0.677	0.250-2.240	1.802	0.670-4.847	2.455	0.763-7.901	0.109
TG (triglyceride) (mg/dl)	1.00	1.526	0.835-2.790	0.668	0.312-1.433	0.886	0.523-1.501	0.080
Blood Pressure (mm Hg)	1.00	0.542	0.233-1.259	1.227	0.666-2.261	1.093	0.543-2.200	0.261

Note. ^a^Based on multiple logistic regression analysis, with Quartiles of ASM Mass as Percentage of Body.

Q1: <13.87%, Q2: 13.87-16.39%, Q3: 16.39-20.46, Q4: >20.46.

**Table 5:** **Odds Ratios (OR) for metabolic syndrome factors by quartiles of fat mass as percentage of body**

	**Q1 (n = 148)**	**Q2 (n = 148)**		**Q3 (n = 148)**		**Q4 (n = 146)**		***p*** ^**a**^
	**OR**	**OR**	**95% CI**	**OR**	**95% CI**	**OR**	**95% CI**	
Waist circumference (cm)^a^	1.00	6.243	2.949-13.218	7.529	3.362-16.861	14.589	6.016-35.381	<0.001
Fasting Glucose (mg/dl)	1.00	0.494	0.261-0.934	1.074	0.536-2.151	0.788	0.389-1.597	0.037
HDL-cholesterol (mg/dl)	1.00	1.480	0.383-5.719	1.477	0.436-5.009	1.429	0.410-4.985	0.926
TG (triglyceride) (mg/dl)	1.00	2.577	1.051-6.316	1.599	0.673-3.799	1.615	0.750-3.478	0.207
Blood Pressure (mm Hg)	1.00	1.431	0.789-2.597	1.855	0.769-4.470	0.926	0.386-2.221	0.139

Note. ^a^Based on multiple logistic regression analysis, with Quartiles of Fat Mass as Percentage of Body.

Q1: <23.05%, Q2: 23.05-29.54%, Q3: 29.54-35.11, Q4: >35.11.

## Discussion

This study identified metabolic syndrome risk factors with groups of health living conditions in a nationally representative sample of Korean elderly, and those factors were differentially associated with dietary patterns, body image, appendicular skeletal muscles mass, and fat mass. The elevated blood pressure was strongly associated with dietary patterns. Waist circumference and HDL-cholesterol, and TG were significant factors in body image. Waist circumference was associated with ASM. Finally, waist circumference and fasting glucose were showed significantly associated with fat mass as percentage of body. Song and Joung [17] showed similar results showing fifty percent of the Korean adult population continues to follow a traditional dietary pattern, having beneficial effects with respect to some metabolic abnormalities. However, the high prevalence of low HDL-cholesterol, attributable to a high-carbohydrate diet, should be considered.

In addition, Park and his collegues [18] examined the construct validity of the metabolic risk factors using confirmatory factor analysis and suggested that, among five components, waist circumference, triglycerides, fasting insulin, and blood pressure were potentially useful phenotypic traits defined. Oh also conducted cluster analysis using metabolic risk factor cutoff values for metabolic syndrome in adults that were not applicable to children [19]. They identified a high-risk group found to be strongly associated with inflammatory markers, implying that even though metabolic syndrome shares common traits, dominant traits could lead to metabolic syndrome differentially.

In addition, we found that five metabolic risk factors were differentially associated with groups of health living conditions. The elevated blood pressure level, but not the fasting glucose level, was positively associated with dietary pattern. There is not much evidence for a link between dietary patterns and metabolic syndrome in elderly. O'Sullivan and colleagues [20] examined the effect of carbohydrate intake on metabolic syndrome and its components in Australian adolescents. They revealed that high carbohydrate intake was significantly associated with an increased risk of metabolic syndrome and elevated triglycerides. There was no clear explanation that dietary pattern was linked with blood pressure. However, high carbohydrate intake is related to high triglyceride levels and low HDL-cholesterol, which are typically associated with dyslipidemia in Asian populations [21-23]. Our study provided evidence that Asian elderly also show the same effect with regard to metabolic risk factors by dietary pattern.

Even though the importance of body image and body compositions in the prevention of metabolic disease has been emphasized, only a few studies have investigated the association between specific health-related factors and metabolic syndrome in elderly. A higher level of muscle and lower level of fat mass were associated with a reduced risk of metabolic syndrome in American adolescents [24]. However, those studies did not report on the associations of the individual components of metabolic syndrome. Further longitudinal studies should be conducted to examine the associations of specific dietary factors with individual or grouped metabolic risk factors to better understand the role of diet on the etiology of metabolic syndrome.

There were several limitations to this study. First, it was a cross-sectional study and, thus, no causal inferences could be drawn from it. Second, we obtained dietary intake information via a single 24-hour recall survey, which may not represent usual dietary intakes. However, we categorized subjects into three according to their pattern scores and compared the percentage and odds ratios among the groups. Finally, the definition of metabolic syndrome in elderly has not been well established and, therefore, misclassification is a concern. Nevertheless, this study was the first to characterize metabolic risk factors and to examine the associations of the identified group related health living conditions in a large nationally representative adolescent sample. Thus, our study has important implications regarding the prevention and management of metabolic risk factors in elderly.
